# Oral Cancer Therapy: Policy Implications for the Uninsured and Underinsured Populations

**DOI:** 10.6004/jadpro.2013.4.5.7

**Published:** 2013-09-01

**Authors:** Beth Faiman

**Affiliations:** From Cleveland Clinic, Cleveland, Ohio

Approximately 400 anticancer drugs are currently in development. The National Comprehensive Cancer Network estimates that 25% of the drugs in development will be available in oral formulations (Weingart et al., 2008; Neuss et al., 2013). It is estimated that the trend for specialty anticancer therapy drugs will increase by 20% within the next 3 years (Express Scripts, 2012). Many oral cancer therapies are not available in an IV or parenteral formulation, as no equivalent exists. Examples include lenalidomide (Revlimid) for the treatment of multiple myeloma or myelodysplastic syndrome (Celgene, 2013a) and imatinib (Gleevec) for treatment of chronic myelogenous leukemia (Novartis, 2013).

The terms "uninsured" and "underinsured" have specific definitions. An individual who is uninsured is one who lacks medical insurance coverage. An individual who is underinsured is one who has medical expenses greater than 10% of one’s annual income or health plan deductibles equaling or exceeding 5% of one’s annual income (Nunley, 2008). The increasing concerns regarding uninsured and underinsured individuals correlate to the rising costs of health care, lack of adherence to recommended treatments, and even shorter survival in some instances.

The problems facing uninsured and underinsured individuals have been addressed more and more often in the past few decades. It is hoped that the Patient Protection and Affordable Care Act (ACA) will fill a gap in coverage for some cancer patients. The ACA will expand insurance coverage for uninsured or underinsured individuals, as the ACA will require health insurance at a minimum in each state: the essential health benefit. It is important to note that as of October 1, 2013, Medicare open enrollment begins and will expand insurance coverage to millions of currently uninsured US citizens (Hutchins et al., 2012). Benefits of the ACA and expanded insurance coverage include more access to preventative services and a greater availability of oral cancer therapies (Hutchins, Samuals, & Lively, 2012). Although oral drug coverage will be improved as a result of the ACA legislation, patient out-of-pocket costs for drugs will likely remain an issue for advanced practitioners to consider.

## Benefits of Oral Cancer Therapies

The benefits of oral cancer therapies have been well described. One major benefit is the unique and sophisticated mechanisms of action of newer treatments. Many targeted oral cancer drugs can provide less harm to healthy cells compared with traditional chemotherapeutic agents and are often associated with fewer side effects (Tigue, Fitzner, Alkhatib, Schmid, & Bennett, 2007). In addition, the convenience of at-home self-administration as well as the freedom from IV therapies, hospital admissions for chemotherapy, and more aggressive therapies are significantly desirable advantages (Faiman, 2011). These benefits, however, are counterbalanced by concerns regarding the patient’s ability to remember to take his/her medications and monitor and manage side effects and, of course, the cost of reimbursement. Studies suggest that patients are more likely to remain adherent to oral cancer therapies if the costs of their medications are covered, as compared to patients whose drugs are not covered (Tamariz et al., 2011).

The high prices for oral cancer therapies seen within the past 15 years reflect the manufacturer costs for drug discovery, development, and marketing (Lee & Emanuel, 2008). In 2005, economists estimated that as effective new cancer drugs advance through various phases of clinical trials from bench to bedside, the cost associated with each drug is approximately $1 billion (Adams & Brantner, 2006; Experts in Chronic Myeloid Leukemia, 2013). Targeted drugs contribute to longer life and improved survival rates in many cancers such as multiple myeloma and chronic myelo-genous leukemia so much so that cancer is now considered a chronic condition. In contrast, medications to treat other cancers can cost $80,000 for one treatment cycle, with an incremental 1.2-month benefit (Fojo & Grady, 2009). Select oral therapies and their approved indications are listed in the Table.

**Table 1:**
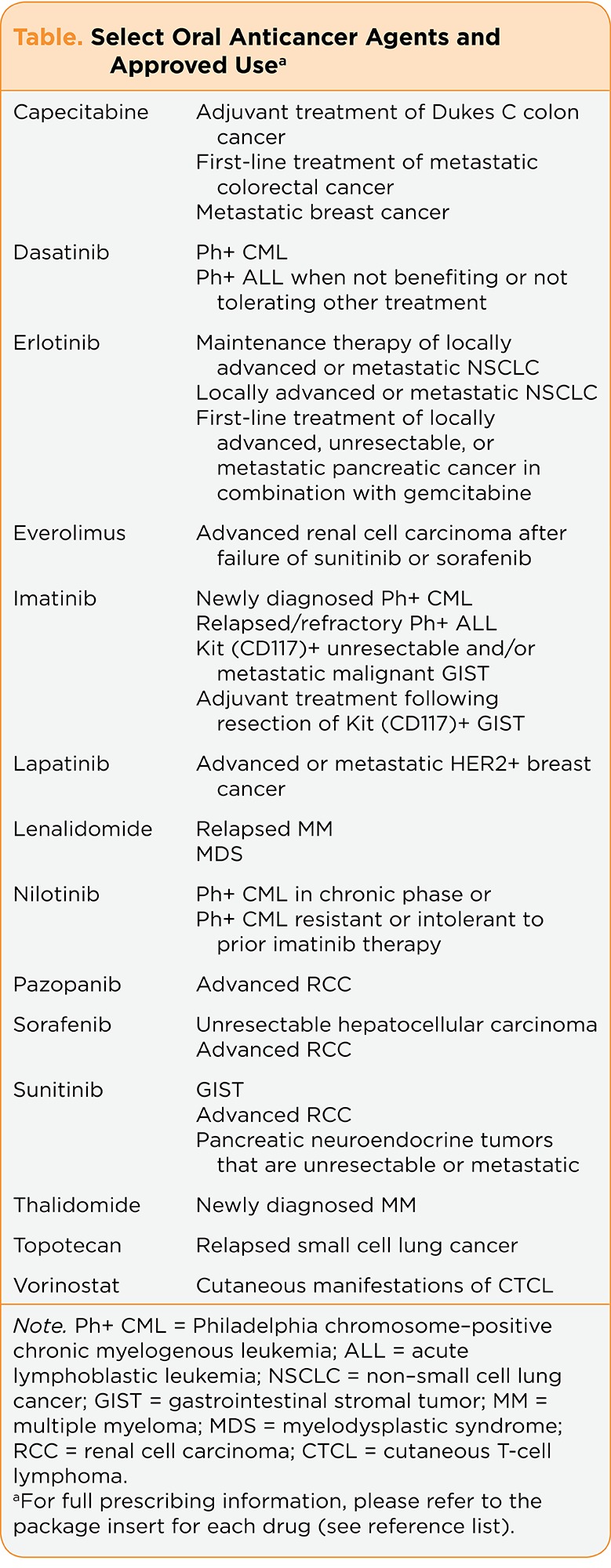
Table. Select Oral Anticancer Agents and Approved Use

## How Medications Are Currently Reimbursed

Reimbursement of medications in the United States is based upon many factors, such as age, type of insurance, and geographic location. Oral and IV cancer-related medications are not reimbursed in the same manner (Neuss et al., 2013). In many states, IV drugs are reimbursed under a health plan’s medical benefit. Orally administered cancer therapies are covered under a health plan’s pharmacy benefit. Thus, a lack of parity in reimbursement of medications exists (Cheema et al., 2012). High copayments for individuals with insurance may prevent patients from receiving potentially life-saving medications required to treat their cancer or may result in patients opting for alternative IV therapies that may be less effective or suboptimal. Many uninsured or underinsured patients will decide to forgo treatment altogether.

The higher cost of oral cancer medications in the United States has led insurance companies to develop a "tiered" system through which prescriptions are currently reimbursed (Lee & Emanuel, 2008). In this payment structure, less-expensive medications are covered at a higher rate with fewer out-of-pocket costs passed down to the patient. This is compared with more expensive drugs (as used in cancer treatment), which will result in higher patient out-of-pocket costs. Medications may be more effective in some instances but carry an enormously high price tag, as patients are charged with 20% to 33% of the total cost. Many oral cancer drugs cost thousands of dollars per month of treatment (Nadler, Eckert, & Neumann, 2006).

The Medicare Part D program is different from the tiered payment structure. Individuals qualify for Medicare Part D if they are over the age of 65 or under the age of 65 with certain disabilities, such as end-stage renal disease or amyotrophic lateral sclerosis. Introduced on January 1, 2006, Medicare Part D provides prescription drug coverage for beneficiaries, but a coverage gap (often referred to as the "donut hole") exists with high-priced drugs (Hede, 2009). The standard drug benefit through Medicare Part D begins with a $275 deductible and a 25% copayment for drugs that cost between $275 and $2510 (Gu, Zeng, Patel, & Tripoli, 2010). Beneficiaries then enter a coverage gap in which 100% of the drug cost is paid until the catastrophic limit of $4050 is reached (Gu et al., 2010).

Oral drug coverage under Medicare is often insufficient to cover total drug costs. In this instance, prescription copayment and patient assistance programs can be accessed through patient advocacy groups such as the Leukemia and Lymphoma Society, the International Myeloma Foundation (IMF), and the Chronic Disease Fund, to name a few. Each group provides financial support to insured patients (which includes Medicare Part D beneficiaries), who must meet specific financial and medical criteria to access these programs. Patients can act as their own advocates and investigate support through the aforementioned organizations or other Internet sites such as www.needymeds.org.

Other options for drug reimbursement include the pharmaceutical companies themselves. All pharmaceutical companies offer some sort of copayment assistance and absorb part or a majority of the copayment costs. Pharmaceutical companies often provide generous reimbursement in an effort to get medication to the patients who need financial assistance the most. Unfortunately, many insured patients will require more financial support to cover the costs of cancer therapy than what is covered through insurance or provided by the pharmaceutical or patient support organizations (Zafar et al., 2013). 

## Economic Analysis

Oncology nurses and advanced practitioners are integral to the policy-making process, which begins with an understanding of cost and economic evaluation. Cost-effective analysis (CEA) is one method to inform us of the effectiveness of an intervention and answer the question "How much benefit do we get with our money?" Two key concepts used in CEA are the quality-adjusted life-year (QALY) and the incremental cost-effectiveness ratio (ICER; Garrison, 2010; National Institute for Health and Clinical Excellence, 2010).

The QALY is designed to measure both the time a new intervention adds to a person’s life and the quality of life the patient experiences in the additional time that is given (Gold et al., 1996; Garrison, 2010). The number 1 represents a year in best possible health and 0 represents the worst possible health, or death. For example, if a person were to be confined to a wheelchair, then the value of that year would be designated as less than 1.

The ICER is the ratio of the difference in mean cost divided by the difference in mean effectiveness. The result is reported as average ICER (in dollars) per QALY. In the United States, an intervention with a QALY dollar amount of < $50,000 is generally considered to be a cost-effective therapy (Hirth et al., 2000). For example, in a 2004 UK study, when oral imatinib was compared to the standard of care (interferon or bone marrow transplant) in patients with chronic myelogenous leukemia, the QALY was £26,180, or approximately $47,120 in 2004 US dollars (Daziel et al., 2005). Using the $50,000 benchmark, the oral imatinib in this example would be considered cost-effective.

Unfortunately, ICER and QALY analyses do not necessarily take into account convenience and factors other than quality of life, such as quantity of life, but ICER and QALY scores are generally reported by economists and researchers to objectify and rationalize the costs of procedures. Advanced practitioners should understand that calculating ICER in cost per QALY is one area in which health-care policy change can be affected. Nurses and advanced practitioners must be aware of economic analysis but can keep in mind that patients should have access to oral cancer therapies regardless of cost and financial constraints. In the argument of free choice and quantity of life, ICER and QALY analyses would not be appropriate as only a minimal increase in survival may be seen with some expensive chemotherapy drugs (Fojo & Grady, 2009).

The importance of cost when evaluating the "best" therapy for a patient with cancer among oncologists has been studied. When surveyed, the majority of oncologists at two major academic centers in Boston stated that cost does not influence the oncologists’ clinical practice, nor should it limit access to "effective" care. The cost-effectiveness thresholds in the physicians surveyed reached $300,000 per QALY, meaning that patients should have access to drugs despite the high costs of therapy (Nadler, Eckert, & Neumann, 2006). As expected, most patients also wanted the best treatment for themselves regardless of cost. However, should we even begin to calculate the cost-benefit ratio in cancer, and is this the same concept as putting a price on life? How does one place a value on human life?

## Policy Change at the State and National Levels

In 2008, Oregon became the first state in the nation requiring insurers to provide equivalent reimbursement for oral and IV cancer-related drugs (Carroll, 2012). As of late 2013, 26 states currently have oral parity legislation (see Figure). Oregon’s change in legislation occurred as a result of lobbying efforts on the part of patients, health-care professionals, advanced practitioners, and advocates. State lawmakers recognized the disparity: Costly medical procedures and IV medications were reimbursed, including some less-efficacious treatments (Carroll, 2012).

**Figure 1:**
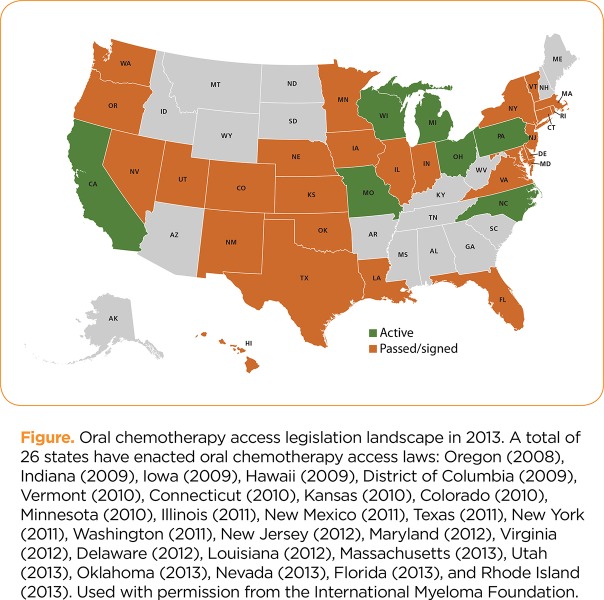
Figure. Oral chemotherapy access legislation landscape in 2013. A total of 26 states have enacted oral chemotherapy access laws: Oregon (2008), Indiana (2009), Iowa (2009), Hawaii (2009), District of Columbia (2009), Vermont (2010), Connecticut (2010), Kansas (2010), Colorado (2010), Minnesota (2010), Illinois (2011), New Mexico (2011), Texas (2011), New York (2011), Washington (2011), New Jersey (2012), Maryland (2012), Virginia (2012), Delaware (2012), Louisiana (2012), Massachusetts (2013), Utah (2013), Oklahoma (2013), Nevada (2013), Florida (2013), and Rhode Island (2013). Used with permission from the International Myeloma Foundation.

A basic tenet of oral parity legislation is that oral cancer therapies do not have an IV equivalent, can be more effective, and yet are not reimbursed at the same rate. The importance of equivocal reimbursement or drug parity has become the basis for federal legislation introduced into Congress in June 2009 as proposed bill H.R. 2746, which requires individual health insurance and group health plans to provide for coverage for oral cancer medications at the same cost as intravenously administered anticancer medications. However, H.R. 2746 was never enacted. In April 2013, the bill was reintroduced as H.R. 1801 (GovTrack.us, 2013). This bill is in its beginning stages but represents the unified voice of patients, nurses, health-care professionals, and patient support organizations.

Patient advocacy groups such as the IMF and the Myelodysplastic Syndromes (MDS) Foundation have provided "statements of principles" in regard to oral parity. These statements emphasize the need for equal insurance coverage, prevention research, innovation of new treatments, expedited US Food and Drug Administration (FDA) approvals, and expanded access to experimental drugs, each vitally important to cancer patients. The high cost of medications affects the uninsured and the underinsured who may be unable to afford potentially life-saving therapies. Oncology nurses and advanced practitioners can partner with organizations such as the IMF and MDS Foundation and use their voice and influence to change parity legislation at state-wide and national levels, which will ultimately benefit our patients.

## Implications for the Advanced Practitioner

There is little argument with the statement that most individuals diagnosed with cancer are faced with financial and psychological burdens. Reimbursement of potentially life-saving oral medications is deficient in most states. If present, parity legislation can lessen the financial burden to patients. Advanced practitioners represent a critical link among patients, support groups, and insurance companies. Thus, it is imperative for the advanced practitioner to understand the role and value of oral cancer therapies and to help remove financial barriers for patients. An understanding of the basics of cost analysis is an essential tool in the struggle to impact health-care and policy change.

Until oral cancer parity has been achieved, the resourceful advanced practitioner will provide guidance for the patient seeking available financial assistance. It is imperative to work in tandem with the social worker, case manager, or navigator to find financial assistance for medications. This may also include overall financial assistance to offset medical, living, and transportation expenses. Numerous patient support (groups as mentioned in this article) can provide various degrees of assistance depending on the patients’ finances; however, patients should be encouraged to act as their own best advocates to seek individual funding where it can be found.

## Conclusion

Oral and IV anticancer medications are necessary for the treatment of cancer. In many cases, it is unreasonable (e.g., clinically inferior, more toxic) for a patient to receive IV therapy instead of oral therapy. Intravenous medications are often reimbursed without substantial out-of-pocket costs to patients, but oral medications may not be reimbursed at all or may be associated with excessive copays. Some patients may lack the insurance or resources to even consider the copayments required to proceed with the recommended course of treatment.

Oncology nurses and advanced practitioners are in a unique position to identify barriers to health-care policy, identify stakeholders, and become change agents within their hospital or organization and at the local or national level. Understanding the concepts of ICER and QALY used by economists and policy makers to argue for or against a therapy is important. Partnering with patient and nursing advocacy groups is of equal importance and can serve as a starting point to address the need for equality in reimbursement among patients with cancer. In this era of health-care reform, we are responsible to use our voices to implement change and advance health-care policy.
